# Evaluation of semi-distributed hydrological models performance in borkena watershed; upper awash basin, Ethiopia

**DOI:** 10.1016/j.heliyon.2023.e18030

**Published:** 2023-07-07

**Authors:** Geteneh Teklie Alemu, Mamaru Moges Ayalew, Berhanu Sinshaw Geremew, Bayu Geta Bihonegn, Kassa Abera Tareke

**Affiliations:** a Water Resource and Irrigation Engineering Department, KIoT, Wollo University, Ethiopia; b Faculty of Civil and Water Resource Engineering, BiT, Bahir Dar University, Ethiopia; c Department of Civil and Environmental Engineering, University of Gondar, Ethiopia

**Keywords:** Borkena, Semi-distributed hydrological models, Model comparison, Infiltration excess

## Abstract

Flood is one of the most significant disasters in human life and economic destruction. To challenge this disaster, the use of models is very important to predict the magnitude and impact of river flow and to find a solution of the problems. This research is aimed to compare the performance of semi-distributed hydrological models in the Borkena watershed. The selected semi-distributed hydrological models were soil and water assessment tool (SWAT), hydrological engineering center-hydrologic modeling system (HEC-HMS), hydrologiska byråns vattenbalansavdelnin (HBV), and parameter efficient distribution (PED). The models were calibrated from 1999 to 2009 and validated from 2010 to 2015 using daily data. Based on validation results; The Nashsutclif (NSC) output of the SWAT, HEC-HMS, HBV, and PED models were 0.68. 0.66, 0.65, and 0.65, coefficient of determination (R^2^) 0.69, 0.67, 0.71, and 0.70, percentage of bias (PBIAS) −6.5, 0.6, 27.34, and 10.28, and root mean square error (RMSE) 14.24, 17.45, 17.63 and 0.91, respectively. Based on the models' performance results in Borkena watershed, the first effective model was SWAT and the second one was HEC-HMS. The HBV and PED models took third and fourth places respectively. The overall results show that the two infiltration excess models (SWAT and HEC-HMS) were performed in a better way than the two saturation excess models (HBV and PED) on this watershed. Therefore, according to the model output, the Borkena watershed is an infiltration excess area.

## 1 Introduction

Different hydrological phenomena have been studied to determine the climate variations on the globe [1]. Recently, various hydrological models have been developed around the world to study the effects of climate change and soil properties on hydrology and water resource systems [2]. Each model differs from the others in terms of the parameters considered, the formulas developed, and the assumptions made. The input data used by the different models are precipitation, air temperature, soil properties, topography, land use, land cover/vegetation, hydrogeology, and other physical parameters [3].

A model is a simplified representation of a real system and is primarily used to predict system behavior and understand various processes [4,5]. Hydrological models are composed of several parameters that have direct or indirect impacts on runoff [6]. These models are very essential to predict the magnitude of river flow [5,7]. This critical aspect of hydrological modeling is used to quantify the effects of flows resulting from different precipitation models with other physical processes. In addition, modeling in hydrology has diversified from the primary task of runoff prediction to a wide range of applications [8,9].

Due to changes in the natural environment, caused by human intervention and the effects of global climate change, floods have recently become a more frequent and unpredictable problem [10] Urbanization and changing demographics in river floodplains have resulted in communities being increasingly exposed to flood risk. Developing countries, especially in Africa, are facing this type of disaster due to deforestation and a lack of awareness on land use mechanisms [11,12]. On the other hand, developed countries, which have much more built-up areas and infrastructure, are also exposed to huge economic losses when the flood is not properly managed [13,14]. Therefore, sufficient and reliable flood forecasting and proper planning of its protection works as mitigation measures are the best solution to deal with this type of disaster. However, this requires an adequate understanding of the hydrological and hydrodynamic processes of the river and the associated catchment characteristics [15].

Currently, many rainfall-runoff models are used by researchers and practitioners, but their applications depend greatly on the objectives for which the model is undertaken [16,17]. Many precipitation and runoff models are used solely for research purposes to understand the hydrological processes controlling a real system, but some models are also used for simulation and forecasting purposes. Hydrologists use these models to estimate flood frequency, and routing pupose. This helps decision-makers and planners to develop mechanisms to control flooding and use resources efficiently [18] Nowadays, many semi-distributed hydrological models have been invented. The simultaneous use of these models at once is very difficult due to time and other constraints. In this study, four semi-distributed hydrological models were selected. These are the SWAT and HEC-HMS (two infiltration excess) and the HBV and PED (two saturation excess) models.

The advantage of the SWAT model is that it is easily available online and allows the modeling of surface and groundwater quantity and quality. It is a comprehensive model that integrates environmental processes at the surface and in the channels. The main weakness of the model is the non-spatial representation of the HRU in each sub-catchment. As a result, the model has been kept simple and supports applications for each watershed. Another limitation of the model is that it does not allow the simulation of multicultural plant communities, as is the case in organic agriculture, grasslands, and forests, as it was originally developed for monocultures [19,20].

The HEC-HMS model is very popular and is adopted in many hydrological studies due to its ability to simulate short- and long-term runoff and its ease to use. This is an integrated modeling tool for all hydrological processes in catchment systems. The model is used to know rainfall loss, direct runoff, and flood routing purpose [21]. The limitation of this model is that it does not use data from land and landform files like the soil and water assessment tool.

The HBV model was chosen because of its relatively low input data requirements. Over the past few decades, the model has demonstrated its flexibility and robustness in solving water resources problems, and it is used for a wide range of applications in different versions in many studies and projects around the world. It is a lump-sum, conceptual model that operates at daily time steps and simulates the discharge using daily precipitation and potential evapotranspiration as inputs. The model consists of three main elements: snow accumulation and melting, consideration of soil moisture and its response, and river routines. It uses sub-catchments as the primary hydrological units, an area-altitude distribution, and a rough land use classification. The model simulates daily flow based on daily precipitation, temperature, and potential evapotranspiration time series [22].

The other selected model in this research was the PED model. This model categorizes saturated, degraded, and downslope areas of the catchment and considers the mutual calibration of runoff parameters. However, the boundaries of the model were calibrated manually. One of the lidmitations of this model is that it has so far relied on manual calibration and is very limited in its ability to perform sensitivity or uncertainty analyses [23].

In practice, it has been found that people who have settled near the Borkena river and produce there are affected by flooding (have been flooded). In the study area, Kombolcha, Harbu, and Kemsie are flood-prone (vulnerable) areas. For example, during the 2006 floods, many buildings were destroyed in Kemsie, and in 2009 many people were also displaced from their survival areas in Kombolcha. Another problem in the selected catchment is the lack of observed flows, except at the outlet point which requires other techniques to predict the discharge in the study area.

The main objective of this research is to evaluate the performance of the semi-distributed hydrological models (SWAT, HEC-HMS and HBV, and PED) in the Borkena watershed. To achieve it, the specific objectives were (1) to estimate the discharge of the river in the Borkena watershed using the selected semi-distributed hydrological models, (2) to compare the efficiency of the selected models using the predicted and observed discharges at the outlet point. Achieving this objective is very important for the construction of certain water-related structures (such as dams, spillways, dikes, reservoirs, etc.) in the selected regions. Planners and decision-makers can use effective models as techniques to predict floods in the selected watershed. By using the most compatible models in the selected region, any governmental or non-governmental organization can use flood prediction methods without observing the discharge at any point of the river in the catchment. Therefore, this research is crucial to understand the best models compatible with the selected region to estimate future floods and indirectly save lives and resources lost due to floods in the selected region.

## 2 Materials and methodology

### 2.1 Description of the study area

**Fig. 1:**
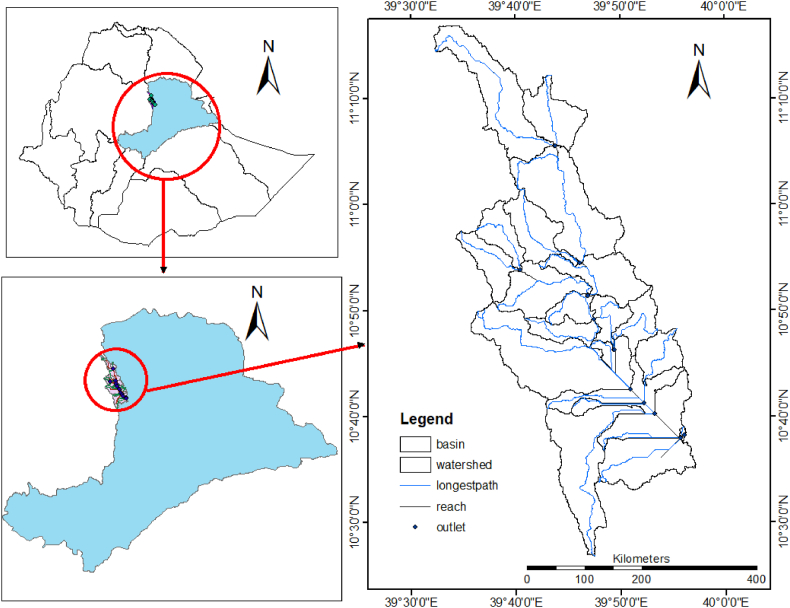
Location of the study area.

The Borkena watershed is located in the South Wollo and Oromia Special Zones in the eastern parts of the Amhara region, Ethiopia. The watercourse of the catchment begins at Mount Tosa, which is near the town of Desie town and forms the upper part of the Awash river basin. The outlet point from the study area is located near Kemsie town, at 10° 38’ north latitude and 39° 56’ east longitude. The total area of the catchment is 1669 km^2^ (Fig. 1).

The topography of the watershed is highly undulated and the elevation varies from 1378 m to 3499 m above mean sea level. From an agroecological point of view, the watershed is under the Woina Dega conditions. The rainfall in the selected catchment has a bimodal characteristic, where the hydrographs increase twice a year. From February to April, it increases slightly and decreases from April to June. Afterward, from June to the end of August, it also increases and decreases again from the beginning of September to February. The average daily temperature and average annual precipitation were 20.45^o^c and 1181 mm, respectively.

### 2.2 Data acquisition

The main input data for this study were meteorological, hydrological, digital elevation model (DEM), land use/land cover, and soil data. These data were obtained from the Ethiopian Meteorological Agency, the Ministry of Water, Irrigation and Energy, and the basin authorities. The meteorological and hydrological data ranges were from 1999 to 2015, and the digital elevation model, land use/land cover, and soil data were also acquired from the agencies in 2011. The types, sources, and extent of data used for this study were presented below (Table 1).

**Table 1:** Hydrometeorological and spatial data.

No.	Data types	Data sources
1	Meteorological data	Ethiopian Meteorology Institution, Bahir Dar branch
2	Hydrological data	Ministry of Water, Irrigation & Energy, Addis Ababa
3	Digital elevation model data	Abbay Basin Authority Office, Bahir Dar branch
4	Land use/Land cover data	Abbay Basin Authority Office, Bahir Dar branch
5	Soil data	Ministry of Water, Irrigation & Energy

### 2.3 Available meteorological data and stations coverage area

**Fig. 2:**
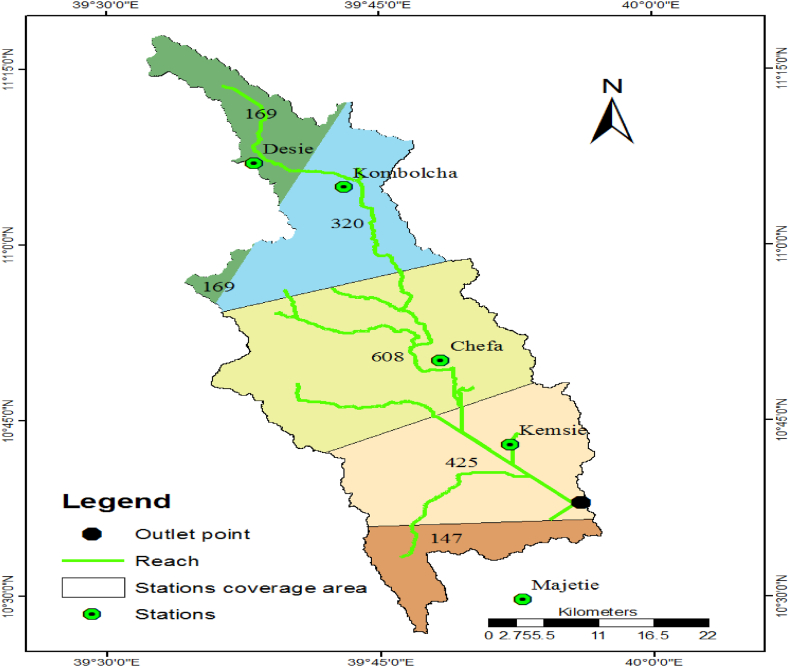
Stations area coverage by Thiessen polygons method.

There are many meteorological stations in the Borkena watershed. But, most of those have incomplete data to conduct research works. From these stations, Dessie, Kombolcha, Cheffa, Kemsie, and Majetie were selected. The Thiessen polygon method was used to estimate the coverage area of each station corresponding to the outlet point of the Borkena river at the swamp (Fig. 2). The types of meteorological data were available and the coverage area of the selected stations were shown below (Table 2).

**Table 2:** Available meteorological data of stations and the class name.

Station name	Precipitation data	Temperature data	Relative humidity data	Solar radiation data	Wind speed data	Stations Class name	Stations coverage area (km^2^)
Desie	Yes	Yes	No	No	No	III	169
Kemsie	Yes	Yes	No	No	No	III	424
Cheffa	Yes	Yes	Yes	Yes	Yes	I	608
Kombolcha	Yes	Yes	Yes	Yes	Yes	I	320
Majetie	Yes	Yes	Yes	Yes	Yes	I	147

### 2.4 Software and materials

The SWAT, HEC-HMS, HBV-light, and PED softwares were mainly used to conduct this research. In addition, other supportive models were used to prepare the input data and the written document. Those software were ArcGIS 10.1, SWAT-CUP 2012, Arc Hydro Tool, HEC-GeoHMS, EndnoteX7, WGEN, EXLSTAT2014, UTM converter, etc. The applications of these softwares have been described below (Table 3).

**Table 3:** Applications of utilized softwares.

No.	Name of the softwares	Applications
1	ArcGIS 10.1	**To support the delineation and export of maps and results intended for SWAT, SWAT -CUP 2012, Arc Hydro Tool, and HEC-GeoHMS softwares.**
2	Mendeley-Desktop-1.19.4	**For citations and references purpose of the research.**
3	HEC-HMS	For discharge prediction
4	EXLSTAT2014	**For data quality analysis.**
5	HBV-light	For discharge prediction
6	SWAT	**For delineation of watershed and simulation of discharges.**
7	PED	For discharge prediction
8	SWAT -CUP 2012	**For sensitivity analysis, calibration, and validation of the runoff simulated by the SWAT software.**
9	WGEN	**To prepare the meteorological input data for the SWAT model in a compatible form.**
10	UTM converter	To prepare the georeferencing points for the localization of stations and output points.

### 2.5 Model performance evaluation techniques

The best-known parameters for evaluating model performance are the coefficient of determination (R^2^), root mean square error (RMSE), Nash-Sutcliffe simulation efficiency (NSE), and percentage of bias (PBIAS) [23,24]. In this study, these statistical methods were applied to evaluate the model performance evaluation technique and selected the best semi-distributed hydrological model.

Coefficient of determination (R^2^); - It estimates the combined dispersion compared to the individual of the observed and predicted series and shows the interdependence (correlation) between the results. If the R^2^ value approaches unity, the flow is effectively predicted by the model.

Root mean square error (RMSE): This value indicates the size of the error between the observed and predicted data values. When the RMSE value tends towards zero, the model is effectively simulating the flow.

Nash-sutcliffe simulation efficiency (NSE): estimates the predictive ability of the model by comparing the observed flows. This efficiency is good when the value is close to unity.

Percentage of bias (PBIAS): This value measures the average tendency of the simulated data to be larger or smaller than the observed data. The optimal value for PBIAS is 0. Positive and negative values indicate an underestimation and overestimation of the models respectively.

### 2.6 Methodology of the research

The primary input data for the simulation of the Hydrological Engineering Center-Hydrologic Modeling System (HEC-HMS) software were digital elevation models (DEM). By using it, the Arc hydro tool extension from ArcGIS software generated several datasets that collectively describe the drainage pattern of the catchment. Initially, the raster analysis was performed in the terrain process to generate a DEM manipulation, completing data on flow direction, flow accumulation, flow definition, flow segmentation, catchment grid delineation, polygon process, drainage line process, slope, and finally catchment process. Once a vector representation of the catchments and drainage lines was developed, the map was used for the HEC-GeoHMS model as input data.

The input data for the HEC-GeoHMS software were the Arc Hydro tools results, land uses land cover (LULC), and soil map. The model is used to extract model-specific information for the HEC-HMS model. In this model, based on the five meteorological stations, the watershed was merged into five sub-catchments. Finally, the hydrological model system legend, scheme, coordinates, shape file background, catchment model file, and weather model file (gauge weight) were generated in HEC-GeoHMS. With this information diversion, junction, reservoir, sink, source, sub-basin, and hydrological modeling system links were computed. All these were the input data for the HEC-HMS model. Using this initial data together with the meteorological and hydrological data, the HEC-HMS simulation was undertaken. Subsequently, the sensitive parameters were identified and the results of the calibration and validation were analyzed.

The initial input for the Soil and Water Assessment (SWAT) model is DEM data. The watershed delineation tool allows the user to delineate the watershed and sub-watersheds using the imported DEM. Flow direction and accumulation were the concepts used to define the river networks, and the hydrological response unit (HRU) was analyzed. Land use/land cover, soil, and slope were calculated from this unit. The land use/land cover and soil data were processed separately for the land cover grids and the soil grids using reference tables. The comparison of these two designations then allowed the reclassification of LU/LC and soil data with several slopes. In addition to the HRU analysis, the meteorological data was also prepared in a form compatible with the SWAT simulation by using the weather generator software (WGEN). Using these simulated and observed hydrological data, SWAT-CUP analyzed all final works from the SWAT model. The identification of sensitive parameters, calibration, and validation process were performed by the SUFI2 algorithm of the SWAT-CUP software. SUFI-2 was also used for multi-site and multi-variable analyses. Therefore, it searches for the best-known measurement data with the lowest possible uncertainties [25].

The input data for the Hydrologiska Byråns Vattenbalansavdelnin (HBV) model were precipitation, temperature, discharge, and evapotranspiration data, which were prepared by using Excel. These data were processed by year, month, and day, transferred to a notepad, and imported into the software. The sensitive parameters were then determined and the calibration and validation processes were also completed.

The parameter Efficient Distribution (PED) model uses meteorological data as an input that predicts the discharges corresponding to the observed one. The sensitive parameters were identified and also the calibration and validation analysis was computed.

**Fig. 3:**
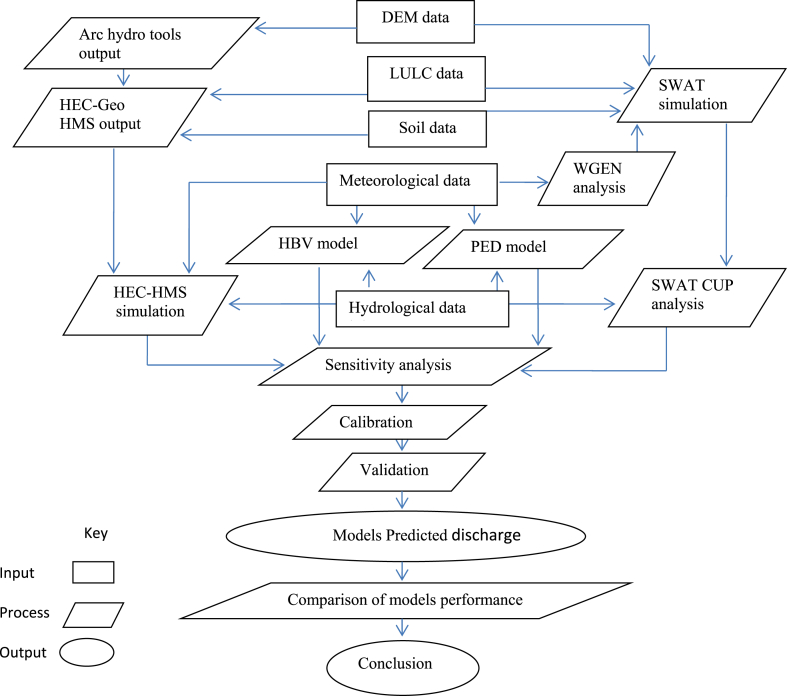
Conceptual framework of the research.

Finally, the flow prediction efficiency of the four semi-distributed hydrological models was compared and the best-suited one from the selected river basin was identified. The general framework of the study is presented below (Fig. 3).

## 3 Results and discussion

### 3.1 Data quality analysis

The best-known techniques used to fill in missing data are the arithmetic mean and the normal ratio method. The normal ratio method is used when the addition and subtraction of the 10% of the normal annual data of the missed station are greater or less than the normal annual data of the other stations and in other ways the arithmetic mean method is used. In this study, these two methods were used to fill in the missed data.

The latest model for testing data quality is the XLSTAT software. This is an extension of excel and can perform any test on time series data. From the XLSTAT software, the Grubbs test is known to perform the outlier test. This method detects one outlier at a time. This outlier is removed from the data set and the test is iterated until no more outliers are found. However, multiple iterations change the detection probabilities, and the test should not be used for sample sizes of six or less, as it often flags most points as outliers [26]. The homogeneity test was also performed with the XLSTAT software. This software considers H_0_: there is no outlier in the data and Ha: there is a minimum/maximum value as an outlier. If the calculated p-value is above the alpha significance level a = 0.05, the null hypothesis H0 cannot be rejected.

#### 3.1.1 Outlier and homogeneity test results

The outlier test was performed for the annual maximum precipitation data of each station during the selected periods. The software calculates the observed, critical, and two-sided Grub p-values. If the percentage of the two-sided p-value is greater than the constant alpha value of 5% (a = 0.05), there is no outlier, otherwise, there is an outlier and the line data should be checked. The results of the outlier tests for the five meteorological stations are presented in the following table (Table 4).

**Table 4:** Grubbs two-tailed outlier test results of meteorological data.

Stations	Desie	Kombolcha	Cheffa	Kemsie	Majetie
G (Observed value)	1.679	1.826	1.926	1.864	1.777
G (Critical value)	2.622	2.622	2.622	2.622	2.622
p-value (Two-tailed)	0.594	0.964	0.726	0.866	0.903
alpha	0.05	0.05	0.05	0.05	0.05

The two-tailed p-values for the Desie, Kombolcha, Cheffa, Kemsie, and Majetie stations, expressed as a percentage, were 59.4%, 96.4%, 72.6%, 86.6%, and 90.3% respectively. At all stations, the p-values were greater than 5% for two variables. Therefore, the null hypothesis H0 cannot be rejected.

As for the precipitation test, for the discharge outlier, the annual maximum values were also selected. The calculated two-tailed p-value was 43.6%, which was above the alpha significance level of a = 5%. There for, one cannot reject the null hypothesis H0. The outlier test results of the hydrological data were described in the following table (Table 5).

**Table 5:** Grubbs two-tailed outlier test of runoff data.

G (Observed value)	2.091
G (Critical value)	2.622
p-value (Two-tailed)	0.436
Alpha	0.05

The homogeneity test was also applied, using the maximum annual precipitation data for each station. The model was instructed to classify the data as homogeneous (H_0_) and non-homogeneous (Ha). Based on the imported data, the model shows the following results (Table 6).

**Table 6:** Homogeneity test of all stations' annual maximum precipitation data.

Stations	Desie	Kombolcha	Cheffa	Kemsie	Majetie
K	38.000	35.000	24.000	30.000	30.000
T	2006	2010	2002	2010	2008
p-value (Two-tailed)	0.196	0.286	0.705	0.439	0.430
Alpha	0.05	0.05	0.05	0.05	0.05

#### 3.1.2Test interpretation

Based on the calculated homogeneity results, the two-tailed p-value of the Desie, Kombolcha, Cheffa, Kemsie, and Majetie stations were 19.6%, 28.6%, 70.5%, 43.9%, and 43.0% respectively. The calculated p-value of the results for each station was above the alpha a = 5% significance level. There for, it indicates that the data is homogeneous.

### 3.2 Identifying sensitive parameters of the models

#### 3.2.1 Identifying sensitive parameters for the SWAT model

There are about 27 parameters for the SWAT model that may or may not significantly affect the magnitude of river discharge [27]. From these parameters, researchers use some very sensitive parameters for the research studies. Before selecting the most sensitive parameters, the model was arbitrarily instructed to run all parameters. Through trial and error, the model indicates the effects of the parameters on the magnitude of river flow in the catchment. The most sensitive parameters were determined based on the t-stat values and the P-values after the analysis was completed. When a parameter is more sensitive, the absolute value of the t-stat result is higher and the P-values are close to zero. Based on this principle, the parameters were excluded from the sensitivity for the selected watershed if the t-stat value of a parameter is zero and the p-value is one. By observing the t-stat and p-value, the thirteen most sensitive SWAT parameters were selected in the Borkena watershed. The selected parameters were described below (Table 7).

**Table 7:** Parameter sensitivity analysis of the SWAT model.

No	Parameter Name	Description of parameters	t-stat	P Value
1	CN2.mgt	SCS runoff curve number	14.396	0.00
2	SLSUBBSN.hru	Average slope length (m)	10.155	0.00
3	SOL_K (.).sol	Saturated hydraulic conductivity (mm/hour)	−8.037	0.00
4	EPCO.hru	Soil evaporation compensation factor	1.298	0.196
5	SOL_AWC.sol	Available water capacity (mm H2O/mm soil)	−1.287	0.199
6	RCHRG_DP.gw	Deep aquifer percolation fraction	−1.153	0.251
7	BIOMIX.mgt	Mixed biomass daily (kg/ha)	0.995	0.321
8	GWQMN.gw	Threshold depth of water for return flow to (mm)	−0.912	0.363
9	SURLAG.bsn	Surface runoff lag time (days)	−0.609	0.543
10	ALPHA_BF.gw	Base flow alpha factor (days)	0.542	0.588
11	REVAPMN.gw	Threshold depth of water "revap" to occur (mm)	−0.344	0.731
12	ESCO.hru	The dry weight of biomass trampled daily (kg/ha)	0.304	0.761
13	GW_DELAY.gw	Groundwater delay (days)	0.055	0.957

The most sensitive parameters for SWAT were the SCS flow curve number, average slope length, and saturated hydraulic conductivity, which have a higher absolute t-stat value of 14.396, 10.155, and 8.037, respectively, and a lower p-value of zero. Other parameters such as soil evaporation compensation factor, available water capacity, deep aquifer percolation fraction, mixed biomass daily, threshold depth of water for return flow, surface runoff lag time, base flow alpha factor, threshold depth of water "revap" to occur, dry weight of biomass trampled daily and Groundwater delay were also sensitive parameters for Borkena watershed.

#### 3.2.2 Parameter sensitivity analysis of the HEC-HMS model

Like SWAT, the HEC-HMS model cannot automatically detect the sensitive parameters. To calculate it, the calibrated values that give the final Nash-Sutcliff efficiency result were very important. Once the calibrated values were known, the sensitivity analysis was applied by manual testing of the parameters for the significance on the magnitude of the predicted discharge. This analysis was done by considering the values of the other parameters as constant, adding and subtracting 50% of the value from the calibrated results, and observing the objective function magnitude. The procedure was applied for each parameter and at the time of sensitivity analysis of one parameter, considering the values of the other value as constant.

**Fig. 4:**
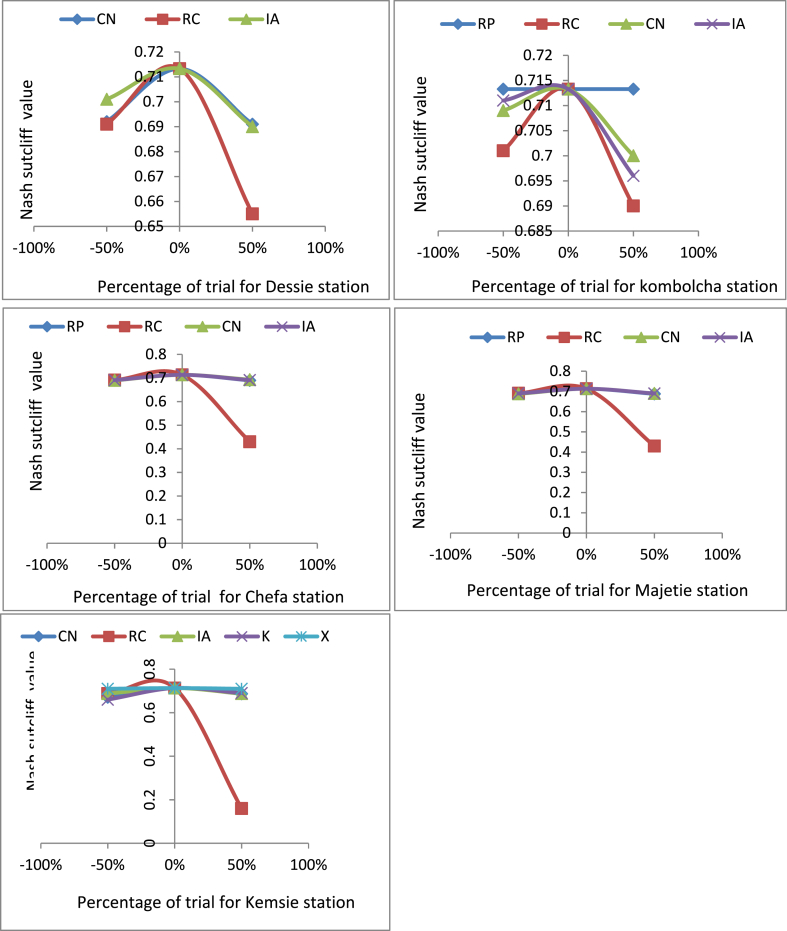
Sensitivity analyses of HEC-HMS model parameters.

Using the HEC-HMS model, the most sensitive parameter was the recession constant (RC) for all sub-basins. Curve number (CN), Initial abstraction (IA), and muskingham-K were also sensitive parameters next to RC. Ratio to peak (RP) and Musgingham-X were less sensitive parameters. The sensitivity of HEC-HMS model parameters were indicated below (Fig. 4).

#### 3.2.3 Sensitivity analysis of the HBV model

The sensitivity analysis procedure for the HBV model was manualy like HEC-HMS. Once the parameters were well calibrated, the sensitivity analysis was identified easily. It was determined by trial and error from the backward and forward values of the calibrated objective function, which is the Nash-Sutcliff efficiency with 10% intervals. Among the nine parameters, the upper zone of the soil layer (UZL), routing length of the weighting function (Maxbase), and field capacity (FC) were the most sensitive parameters than the others. Percolation (Perc) and recession constants (K0, K1, and K2) have small contributions. Other parameters, such as limited potential evapotranspiration (LP) and the non-linearity parameter (shape coefficient) (Beta), do not contribute to the simulation of runoff in the HBV model. The results of the sensitivity analysis of the HBV model were described below (Table 8).

**Table 8:** Sensitivity analysis of HBV model parameters.

percentage	FC	LP	Beta	Perc	UZL	Ko	K1	K2	Maxbase
50%	0.708	0.714	0.714	0.713	0.705	0.71	0.71	0.713	0.708
40%	0.71	0.714	0.714	0.713	0.708	0.711	0.711	0.713	0.709
30%	0.711	0.714	0.714	0.713	0.711	0.712	0.712	0.713	0.711
20%	0.712	0.714	0.714	0.713	0.712	0.713	0.713	0.713	0.712
10%	0.713	0.714	0.714	0.714	0.713	0.713	0.713	0.714	0.713
0%	0.714	0.714	0.714	0.714	0.714	0.714	0.714	0.714	0.714
−10%	0.714	0.714	0.714	0.714	0.713	0.713	0.713	0.714	0.713
−20%	0.714	0.714	0.714	0.713	0.712	0.713	0.713	0.713	0.712
−30%	0.714	0.714	0.714	0.713	0.709	0.711	0.713	0.713	0.711
−40%	0.714	0.714	0.714	0.713	0.706	0.709	0.712	0.713	0.708
−50%	0.714	0.714	0.714	0.713	0.702	0.705	0.711	0.712	0.705

#### 3.2.4 Sensitivity analysis of the PED model

Once the fitted values of the parameters were obtained from the calibrated values, the sensitivity analyses were performed manually. The procedure was applied by checking the value of the objective function (nash-sutcliff efficiency) and continuing the analysis for each parameters. The sensitivity analysis of PED model parameters were described below (Table 9).

**Table 9:** Sensitivity analysis of PED model parameters.

percentage	Saturated area	Degraded area	Hillside area	BS max of GW (mm)	Base flow half-life (t1/2)	Interflow (τ*)	AS Smax (mm)	AD Smax (mm)	AH Smax (mm)
0.5	0.761	0.794	0.363	0.785	0.726	0.785	0.786	0.786	0.79
0.4	0.767	0.785	0.5	0.785	0.742	0.785	0.786	0.786	0.789
0.3	0.772	0.785	0.61	0.785	0.756	0.785	0.786	0.786	0.789
0.2	0.777	0.785	0.695	0.785	0.769	0.785	0.785	0.786	0.788
0.1	0.781	0.785	0.753	0.785	0.779	0.785	0.785	0.786	0.786
0	0.785	0.785	0.785	0.785	0.785	0.785	0.785	0.785	0.785
−0.1	0.788	0.785	0.791	0.785	0.785	0.785	0.785	0.775	0.783
−0.2	0.791	0.785	0.77	0.785	0.778	0.785	0.785	0.763	0.781
−0.3	0.794	0.785	0.724	0.785	0.759	0.785	0.784	0.742	0.779
−0.4	0.795	0.785	0.651	0.785	0.724	0.785	0.784	0.729	0.775
−0.5	0.797	0.785	0.552	0.785	0.665	0.785	0.784	0.706	0.771

### 3.3 Calibrated models result

The four semi-distributed hydrological models used in this study have different parameters. The duration of the calibration data for this study is eleven years with a daily time step starting on 01/01/1999 and ending on 31/12/2009.

#### 3.3.1 Calibration results of SWAT model

**Fig. 5:**
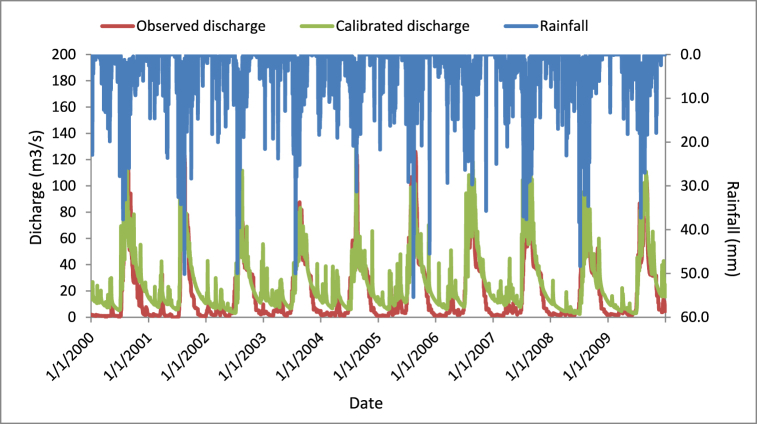
SWAT CUP calibrated discharge graph.

The calibration of the current flow was carried out using the sensitive parameters, which have great significance for the magnitude of the simulated flow. The minimum and maximum values of the selected sensitive parameters for SWAT model were; SCS flow curve number (35–98), Average slope length (10–150 m), saturated hydraulic conductivity (0–2000 mm/h), soil evaporation compensation factor (0–1), available water capacity (0–1 mm of H2O/mm soil), deep aquifer percolation fraction (0–1), mixed biomass daily (0–1 kg/ha), threshold depth of water for return flow (0–5000 mm), surface runoff lag time (0.05–24 day), base flow alpha factor (0–1 day), threshold depth of water "revap" to occur (0–500 mm), dry weight of biomass trampled daily (0–1) and groundwater delay (0–500 day). After many trials and errors, the magnitude of the objective functions, which was the nash sutcliff efficiency, can not change the value. This indicates, at that point the parameter value was the calibrated results. Within ninety-five percent of predicting determinacy, the simulated discharge adopted the observed one in the correct direction, as described below (Fig. 5).

As shown in Fig. 5, the precipitation, calibrated, and observed runoff have well adopted the trends of each other. The last efficiency measurement of the calibrated model, which gave the nash-sutcliff efficiency (NSE), coefficient of determination (R^2^), root mean square error (RMSE), and the percentage of bias (PBIAS), shows that 0.68, 0.695, 16.02 and −21.97. respectively.

#### 3.3.2 Calibration results of the HEC-HMS model

Based on the selected five meteorological stations, five sub-watersheds, nineteen rivers, and junction branches were generated from the HEC-HMS model. The model calibration technique was applied first by instructing it, to operate within any parameter value between the standard ranges [28]. The minimum and maximum values of the selected HEC-HMS model parameters were: curve number (35–98), initial abstraction (0–500 mm), recession constant (greater than 0.00001), and lag time parameters (0.1–500 h), Muskingham K (0.1–150 h) and Muskingham X (0–0.5). The calibration analysis in HEC-HMS was applied by trial and error. Based on the calibrated results, using the nash-sutcliff model efficiency as an objective function, there was a good agreement between the calibrated and observed discharges at the outlet point of the study area.

**Fig. 6:**
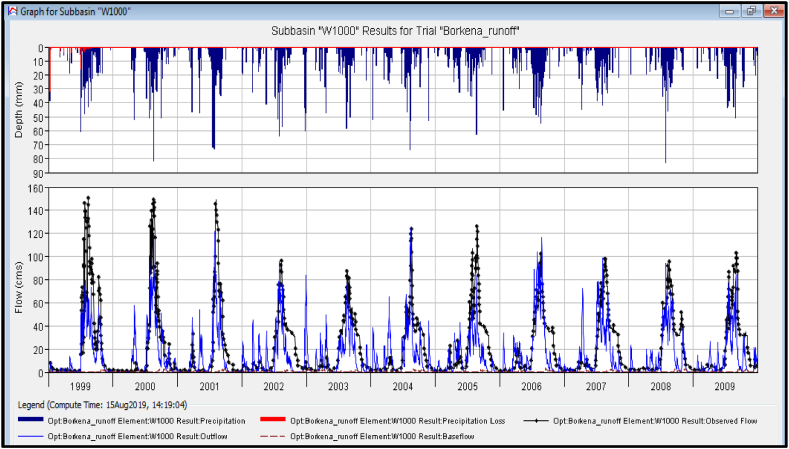
Calibrated discharges graph for the HEC-HMS model.

The best-calibrated value of the HEC-HMS model efficiency was; nash sutcliff efficiency (NSE), coefficient of determination (R^2^), root mean square error (RMSE), and percentage of bias (PBIAS) were 0.713, 0.719, 15.156, and −10.6 respectively. The simulated and observed precipitation time series were given in a good relationship as described below (Fig. 6).

#### 3.3.3 Calibration results of the HBV model

In the hydrologiska byråns vattenbalansavdelnin (HBV) model, there are four routines: snow, soil moisture, response routines, and routing routines. However, there is no snow routine in Ethiopia and this was not included in the parameter analysis. The three routines have nine parameters that can contribute to the magnitude of the simulated discharges. The calibration was initiated via instruction with random numbers from the predefined standard range of parameters [29]. The minimum and maximum values of the HBV model parameters were; the upper zone of the soil layer (0–600 mm), routing length of the weighting function (1–7 days), field capacity (1–100 mm), Percolation (0–6 mm/day), near-surface flow storage constant K_0_ (0.05–0.5 per day), interflow storage constant K_1_ (0.01–0.5 per day), baseflow storage constant K_2_ (0.001–0.1 per day), limited potential evapotranspiration (0.3–1) and the non-linearity parameter beta (1–6).

The calibrated value was obtained by trial and error until the result of the objective function became constant. The test was applied for individual parameters, via plus and minus 50% of the previous values with intervals of 10% while keeping the other parameters as constant. After numerous trials and errors, the calibrated values of the nine parameters of the selected watershed were obtained at zero percent, as described below (Table 10).

**Table 10:** Calibrated HBV model parameters result.

± trials	FC	LP	BETA	PERC	UZL	Ko	K1	K2	MAXBASE
50%	1.5	0.75	1.5	5.25	765	0.1485	0.057	0.0405	9.75
40%	1.4	0.7	1.4	4.9	714	0.1386	0.0532	0.0378	9.1
30%	1.3	0.65	1.3	4.55	663	0.1287	0.0494	0.0351	8.45
20%	1.2	0.6	1.2	4.2	612	0.1188	0.0456	0.0324	7.8
10%	1.1	0.55	1.1	3.85	561	0.1089	0.0418	0.0297	7.15
0%	1	0.5	1	3.5	510	0.099	0.038	0.027	6.5
−10%	0.9	0.45	0.9	3.15	459	0.0891	0.0342	0.0243	5.85
−20%	0.8	0.4	0.8	2.8	408	0.0792	0.0304	0.0216	5.2
−30%	0.7	0.35	0.7	2.45	357	0.0693	0.0266	0.0189	4.55
−40%	0.6	0.3	0.6	2.1	306	0.0594	0.0228	0.0162	3.9
−50%	0.5	0.25	0.5	1.75	255	0.0495	0.019	0.0135	3.25

**Fig. 7:**
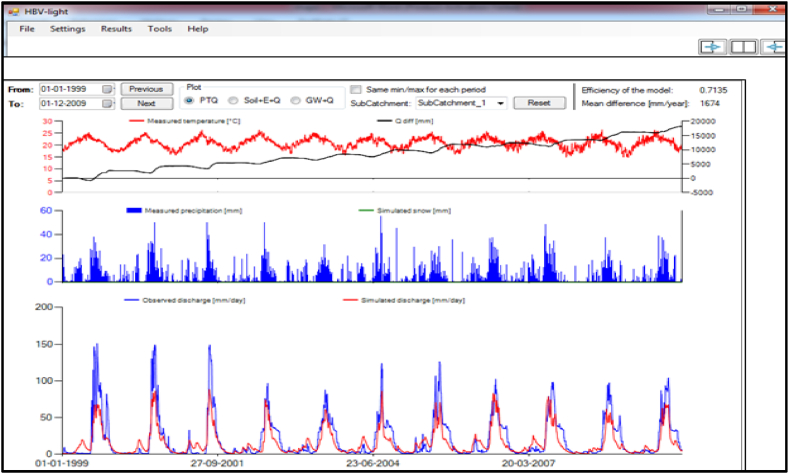
Calibrated discharges of HBV model.

The calibrated HBV model efficiencies such as nash-Sutcliffe efficiency (NSE), coefficient of determination (R^2^), root mean square error (RMSE), and the percentage of bias (PBIAS) were 0.714, 0.81, 15.13, and 22.6 respectively. After many trials and errors, precipitation, predicted and observed discharge have been adopted each other. The final observed and predicted discharges using the objective function of the HBV model have been described below (Fig. 7).

#### 3.3.4 Calibration result of the PED model

The parameter efficiency distribution (PED) model has nine parameters. These parameters, minimum and maximum values, are saturated area (0–100%), degraded area (0–100%), permeable area (0–100%), Smax of saturated area (50–250 mm), Smax of degraded areas (5–2200 mm), Smax of permeable areas (10–300 mm), maximum storage of groundwater (50–500 mm), base flow half-life (50–100 day), and interflow (1–500 day). The saturated area represents the proportion of the watershed that was on the valley floor, the degraded area is the slopes with a limited infiltration capacity through the hardpan, and the hillside area contains percolation rates higher than the daily intensities of precipitation [19,30].

The calibration technique of the PED Model was carried out by trial and error with intervals of 10% compared to the initial test via keeping other parameters with constant. Based on the calibrated parameters, the model shows that the direct flow at the outlet point from the saturated area contains 4.5%, degraded 24.5%, and hillside 71% of the watershed. The hillside area has made a very high contribution to the estimation of discharge in the Borkena watershed. The calibrated values of PED model parameters were described below (Table 11).

**Table 11:** Calibrated parameters value for the PED model.

± trials	Saturated area	Degraded area	Hill area	BSmax of GW (mm)	Base flow half-life (t1/2)	Interflow (τ*)	AS Smax (mm)	AD Smax (mm)	AH Smax (mm)
50%	0.068	0.368	1.065	675	37.5	45	15	3150	22.5
40%	0.063	0.343	0.994	630	35	42	14	2940	21
30%	0.059	0.319	0.923	585	32.5	39	13	2730	19.5
20%	0.054	0.294	0.852	540	30	36	12	2520	18
10%	0.05	0.27	0.781	495	27.5	33	11	2310	16.5
0%	0.045	0.245	0.71	450	25	30	10	2100	15
−10%	0.041	0.221	0.639	405	22.5	27	9	1890	13.5
−20%	0.036	0.196	0.568	360	20	24	8	1680	12
−30%	0.032	0.172	0.497	315	17.5	21	7	1470	10.5
−40%	0.027	0.147	0.426	270	15	18	6	1260	9
−50%	0.023	0.123	0.355	225	12.5	15	5	1050	7.5

**Fig. 8:**
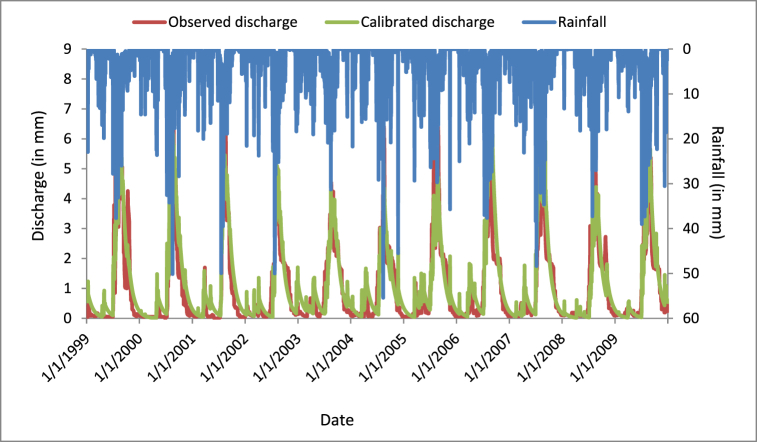
Calibrated discharge graph of the PED model.

The calibration technique was applied by a univariate system, which means that the other parameters are controlled as constant, and the test continues for other parameters. Based on the calibrated analysis, the results of the Nash Sutcliff efficiency (NSE), coefficient of determination (R^2^), root mean square error (RMSE) and percentage distortion (PBIAS) was 0.785, 0.794, 0.68, and −10.74 respectively. The calibrated and observed analysis of the flow trends with its precipitation is described below (Fig. 8).

### 3.4 Validated models results

The accuracy of the calibrated parameters value is checked by validation. Daily data over six years were used for the four semi-distributed hysterological models validation, from 01/01/2010 to 31/12/2015.

#### 3.4.1 Validation results of the SWAT model

**Fig. 9:**
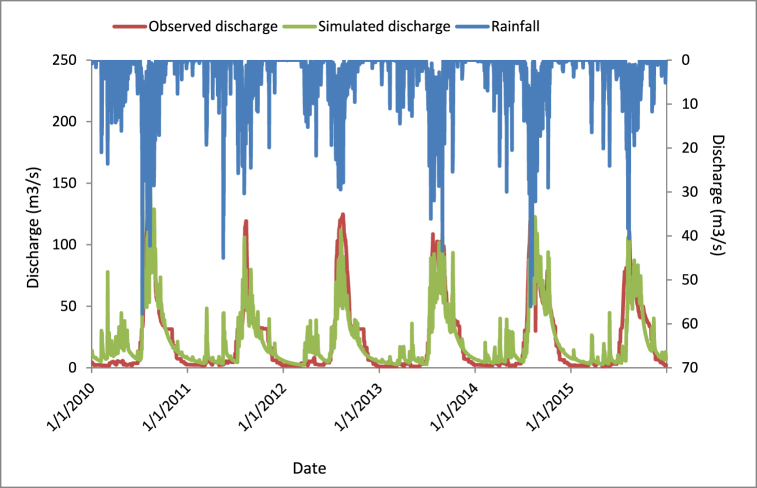
SWAT CUP validated discharge graph.

There is avalidated file which was prepared from SWAT. This file imported into SWAT-CUP and kept the calibrated parameters as constant values and read the objective function. The validation results for nash-sutcliff efficiency (NSE), coefficient of determination (R^2^), root mean square error (RMSE), and percentage of bias (PBIAS) were 0.68, 0.69, 14.24, and −6.5 respectively. At the end of the validation, the precipitation, simulated, and observed discharges had well adopted to each other (Fig. 9).

#### 3.4.2 Validation results of the HEC-HMS model

**Fig. 10:**
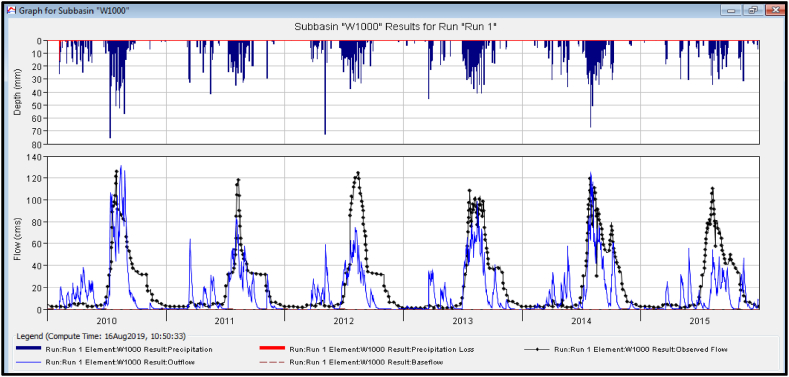
Validated discharges graph of the HEC-HMS model.

After the parameters of the HEC-HMS model had been calibrated, the values were imported directly for the validation data. For this purpose, the value of the calibrated parameters was kept constant and the results were read after the imported values. The validated model efficiency measured on the Borkena watershed for the HEC-HMS model, i.e. Nash-Sutcliff efficiency (NSE), coefficient of determination (R^2^), root mean square error (RMSE), and percentage of bias (PBIAS) were 0.66, 0.67, 17.45 and 0.60, respectively. The trends of the validated HEC-HMS model for precipitation, simulated, and observed discharge were shown below (Fig. 10).

#### 3.4.3 Validation results of the HBV model

**Fig. 11:**
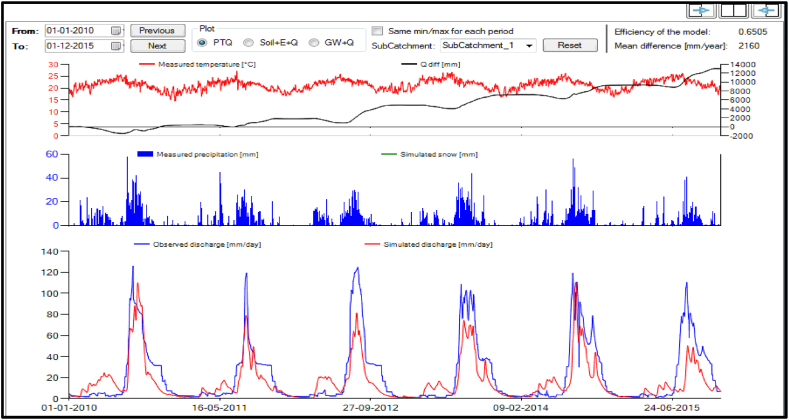
Validated discharge graph HBV model.

The optimized HBV model parameters value were obtained from the calibration. By keeping the calibrated parameters value as constant, the validated HBV model results which were nash-sutcliff efficiency (NSE), coefficient of determination (R^2^), root mean square error (RMSE), and percentage of bias (PBIAS) were 0.65, 0.71, 17.63 and 27.34% respectively. The graph of the simulated and observed discharges with respects to precipitation were described below (Fig. 11).

#### 3.4.4 Validation results of the PED model

**Fig. 12:**
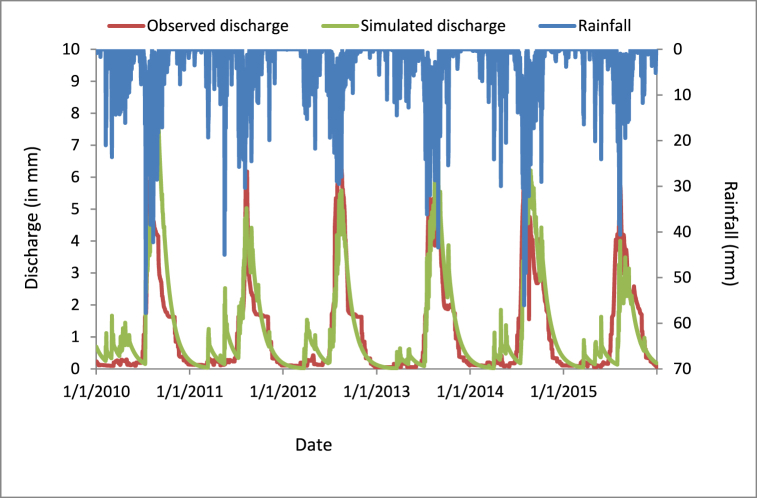
PED model validated discharge graph.

By using the calibrated parameters as constant, and observing objective function, which was nash sutcliff efficiency (NSE), coefficient of determination (R^2^), root mean square error (RMSE), and percentage of bias (PBIAS), were 0.65, 0.70, 0.91, and −10.28 respectively. The validated discharge was well adopted to the observed one with its precipitation, as described below (Fig. 12).

## 4 Conclusion

The four semi-distributed hydrological models applied to compare the capacity for predicting runoff in the Borkena watershed were the soil and water assessment tool (SWAT), the hydrological engineering center-hydrological modeling system (HEC-HMS), Hydrologiska Byråns Vattenbalansavdelning (HBV) and parameter-efficient distribution (PED) models. The criteria for evaluating the performance of the models were the nash-sutcliffe efficiency (NSE), coefficient of determination (R^2^), root mean square error (RMSE), and percentage of bias (PBIAS) for both the calibration and validation purposes. Based on the validation results, the output of nash Sutcliffe efficiency (NSE), SWAT, HEC-HMS, HBV, and PED models was 0.68. 0.66, 0.65, and 0.65, coefficient of determination (R^2^), 0.69, 0.67, 0.71, and 0.70, percentage of bias (PBIAS) −6.5, 0.6, 27.34 and 10.28, and the root mean square error (RMSE) were also 14.24, 17.45, 17.63 and 0.91, respectively.

From the four semi-distributed hydrological models, based on the Nash-Sutcliff efficiency comparison technique, the soil and water assessment tool holds the best executed one on the Borkena watershed. The second-best adjusted results were obtained by the hydrological engineering center-hydrological modeling system. The third and fourth places of the comparison were Hydrologiska Byråns Vattenbalansavdelning and parameter efficient distribution models.

In general, the two infiltration excess models, which were the soil and water assessment tool and hydrological engineering center-hydrological modeling system, had the most accurate results than the two saturation excess models, which were Hydrologiska ByrånsVattenbalansavdelning and parameters efficient distribution models. So, this indicates that Borkena watershed is an infiltration excces area.

Therefore, the government, planners, or decision-makers in/near the Borkena watershed can use the soil and water assessment tool model directly, in the runoff estimation purpose to build certain structures like dams, spillways, reservoirs, etc. There is an observed discharge at the outlet point of the Borkena watershed, but not from other cross sections of the river. So, on this type of challenge in and around the Borkena watershed, it is recommended to use the soil and water assessment tool model for the discharge estimation technique. Any risk takers that may be individual person or organizations can also use this model to carry out appropriate measurements and implementation to solve water-related problems, such as floods and droughts.

One of the limitations of this article was that out of many semi-distributed hydrological models, only four were applied from here. This was done because the time and space required will be very important. The second limitation of this study were, the utilized data was carried out with daily time steps. For more certainty, a comparison with other time series such as hourly, weekly, monthly, annual, etc. have been recommended. The total duration of the research was seventeen years of meteorological and hydrological data. It has been a long time and creates good conditions for the accuracy of forecasted discharges. But for these data ranges, the land use/land cover data were classified in 2011. Since the meteorological and hydrological data have been collected for 17 years, a single land use/cover classification may not be sufficient. Therefore, it is recommended to classify the LULC in two or more periods.

## Author contribution statement

Geteneh Teklie Alemu: Conceived and designed the experiments; Performed the experiments; Analyzed and interpreted the data; Wrote the paper.

Mamaru Moges Ayalew: Conceived and designed the experiments; Performed the experiments.

Berhanu Sinshaw Geremew: Analyzed and interpreted the data; Wrote the paper.

Bayu Geta Bihonegn, Kassa Abera Tareke: Performed the experiments; Analyzed and interpreted the data.

## Data availability statement

Data will be made available on request.

## Declaration of competing interest

The authors declare that they have no known competing financial interests or personal relationships that could have appeared to influence the work reported in this paper.
